# Intraductal Papillary Mucinous Neoplasm (IPMN) of the Pancreas: History, Myths, and Realities Between Past and Future

**DOI:** 10.3390/medsci14030405

**Published:** 2026-07-19

**Authors:** Riccardo Urgesi, Cristiano Pagnini, Maria Carla Di Paolo, Lorella Pallotta, Gianfranco Fanello, Pavlos Antypas, Elio Pietro Perrone, Giuseppe Villotti, Andrea D’Amico, Fernando De Angelis, Maria Giovanna Graziani

**Affiliations:** 1 Gastroenterology Department, S. Giovanni Addolorata Hospital, 00184 Rome, Italy; rurgesi@hsangiovanni.roma.it (R.U.);; 2 Gastroenterology and Endoscopy Unit, Campus Bio Medico, University of Rome, 00128 Rome, Italy; 3 S. Carlo di Nancy Hospital, 00165 Rome, Italy

**Keywords:** intraductal papillary mucinous neoplasm, pancreatic cystic neoplasm, KRAS, GNAS, pancreatic ductal adenocarcinoma, surveillance, adenoma–carcinoma sequence, field defect

## Abstract

Intraductal papillary mucinous neoplasm (IPMN) of the pancreas is among the most clinically relevant and conceptually intricate precancerous lesions encountered in modern gastroenterology. First identified in the early 1980s as a “mucin-producing tumor,” IPMN has since undergone a profound redefinition: from an obscure and poorly classified entity to a well-established precursor of pancreatic ductal adenocarcinoma (PDAC), shaped by characteristic molecular alterations such as KRAS, GNAS, and RNF43 mutations. Over the past two decades, the reported incidence of IPMN has risen sharply, a trend largely attributable to the widespread use of high-resolution cross-sectional imaging rather than a genuine increase in disease prevalence. IPMNs are categorized anatomically into main-duct (MD-IPMN), branch-duct (BD-IPMN), and mixed-type forms and histologically into gastric, intestinal, pancreatobiliary, and oncocytic subtypes, each associated with distinct malignant potential and prognostic implications. International consensus guidelines (Sendai 2006; Fukuoka 2012; Fukuoka revision 2017; Kyoto 2024) have progressively refined strategies for risk stratification and surgical decision-making. Nevertheless, significant debate persists regarding optimal surveillance intervals, thresholds for resection, and the management of low-risk branch-duct lesions. This review offers a comprehensive and critically evaluated synthesis about IPMN, spanning its historical recognition, molecular pathogenesis, epidemiology, clinical manifestations, diagnostic evaluation, pathological features, differential diagnosis, surveillance paradigms, long-term complications, associated conditions, therapeutic options, and future directions. Particular attention is given to longstanding “myths” that have influenced clinical practice and to emerging “realities” grounded in contemporary molecular and clinical evidence. Our aim is to provide physicians with a clear and updated framework for navigating the complexities of IPMN management in current practice.

## 1. Introduction

The identification of intraductal papillary mucinous neoplasm (IPMN) as a distinct clinicopathological entity has profoundly changed the vision of pancreatic tumorigenesis. IPMN is defined as a grossly visible (typically ≥5 mm), mucin-producing intraductal epithelial neoplasm arising within the main pancreatic duct (MPD) or its side branches. It is distinguished from mucinous cystic neoplasms (MCNs) by the absence of ovarian-type stroma and by its characteristic communication with the pancreatic ductal system [1,2]. Importantly, IPMN follows a well-established adenoma–dysplasia–carcinoma sequence, progressing from low-grade dysplasia to high-grade dysplasia (HGD) and ultimately to invasive carcinoma. This stepwise evolution positions IPMN as a pivotal opportunity for early interception of pancreatic ductal adenocarcinoma (PDAC), one of the most lethal malignancies globally [3].

The historical trajectory of IPMN is marked by evolving terminology, shifting classification schemes, and enduring diagnostic ambiguities. In the early 1980s, Ohashi and colleagues first described what would later be recognized as IPMN under the label “mucin-producing cancer of the pancreas.” Over subsequent years, the lesion was variably referred to as “mucinous ductal ectasia,” “intraductal mucin-hypersecreting neoplasm,” and “villous adenoma of the pancreatic duct,” reflecting the conceptual uncertainty surrounding its nature. Only in 1996 did the World Health Organization (WHO) formalize the unified designation “intraductal papillary mucinous neoplasm” [4,5]. This early terminological heterogeneity, one of the persistent “myths” in the field, contributed to inconsistencies in reported natural history, malignant potential, and clinical outcomes.

Over the past three decades, research on IPMN has expanded dramatically. A bibliometric analysis covering 1990–2021 identified 879 eligible publications, highlighting a transition from foundational descriptions and basic science toward themes centered on management, prognosis, and risk stratification [6]. The International Association of Pancreatology (IAP) has played a central role in shaping clinical practice, issuing consensus guidelines in 2006 (Sendai); 2012 (Fukuoka); 2017; and, most recently, 2024 (Kyoto), each iteration refining algorithms for risk assessment and surgical decision-making [7]. Despite these efforts, the guidelines remain consensus-driven rather than evidence-based, and notable discrepancies persist among the IAP, the American Gastroenterological Association (AGA), and the European Study Group on Cystic Tumours of the Pancreas [8,9,10]. These divergences underscore the ongoing uncertainty clinicians face when managing this heterogeneous disease.

At the heart of IPMN management lies a fundamental tension between two competing imperatives. The first is preventing progression to PDAC through timely surgical resection. The second is avoiding unnecessary pancreatic surgery in patients whose lesions may never undergo malignant transformation, that represents a relevant concern, given that such procedures are associated with substantial morbidity and non-negligible mortality. This dilemma is further complicated by the concept of IPMN as a “field defect,” wherein the entire pancreas exhibits a predisposition to neoplastic change. As a result, even after curative resection, patients require lifelong surveillance to monitor for metachronous lesions or recurrent disease [11].

The present review seeks to navigate these complexities by synthesizing current evidence into a coherent framework that distinguishes well-established realities from enduring myths. Through this lens, we aim to clarify the evolving understanding of IPMN biology, refine risk stratification strategies, and highlight the critical areas where future research must advance to optimize patient outcomes.

## 2. Pathogenesis: Molecular Drivers and the Adenoma–Carcinoma Paradigm

### 2.1. The KRAS–GNAS Axis as a Foundational Driver

The molecular pathogenesis of IPMN is anchored by two early and characteristic driver mutations: activating alterations in KRAS (most commonly at codon 12) and in GNAS, typically involving the R201C/H hotspot. A landmark whole-exome sequencing study demonstrated that GNAS mutations occur in approximately 41% of IPMNs yet are virtually absent in conventional pancreatic ductal adenocarcinoma (PDAC), establishing GNAS as a molecular hallmark of IPMN-derived neoplasia and a key discriminator from de novo PDAC [12]. GNAS encodes the stimulatory G protein alpha subunit (Gαs). Mutations at R201 abolish intrinsic GTPase activity, locking Gαs in a constitutively active state and driving persistent cyclic AMP (cAMP) accumulation with downstream activation of protein kinase A (PKA) signaling [13]. These events promote mucin hypersecretion, one of the defining phenotypic features of IPMN, as well as proliferative signaling within the ductal epithelium.

KRAS mutations, present in the majority of IPMNs, activate the canonical RAS–MAPK pathway and represent the earliest initiating event shared with conventional PDAC. The co-occurrence of KRAS and GNAS mutations is particularly characteristic of gastric and intestinal IPMN subtypes, and their synergistic effects likely underpin the papillary, intraductal growth pattern that distinguishes IPMN from the flat architecture of pancreatic intraepithelial neoplasia (PanIN) [14].

Emerging evidence suggests that these mutations may also serve as non-invasive biomarkers. A recent pilot study detected KRAS and GNAS mutations in circulating cell-free DNA (cfDNA) and circulating epithelial cells of patients with IPMN, with GNAS mutations more frequently observed in resected cases than in those under surveillance—an observation that supports their potential utility in liquid biopsy-based risk stratification [15]. Further insight into GNAS-driven tumorigenesis comes from the observation that McCune–Albright syndrome, caused by germline GNAS mosaicism, predisposes to IPMN-associated pancreatic cancer, providing rare but compelling clinical evidence of GNAS-mediated susceptibility [16].

### 2.2. Molecular Events Associated with Progression

Progression from low-grade dysplasia to high-grade dysplasia (HGD) and ultimately to invasive carcinoma reflects the sequential accumulation of additional genetic alterations. Among these, RNF43—a negative regulator of Wnt signaling—is frequently mutated in IPMN and appears to be enriched in lesions exhibiting higher-grade dysplasia, implicating it in the transition toward malignancy [14].

Mutations in TP53 represent a pivotal event in malignant transformation. Next-generation sequencing (NGS) studies have shown that TP53 alterations can be detected in pure pancreatic juice, raising the possibility of liquid biopsy-based surveillance for high-risk lesions [17]. Loss of key tumor suppressors such as SMAD4 and CDKN2A/p16 characterizes the late stages of progression and mirrors the molecular evolution observed in conventional PDAC [14].

KRAS mutations have also been examined in relation to clinicopathological features. Chang et al. demonstrated subtype-specific associations, supporting the potential role of KRAS mutational status as a biomarker for risk stratification [18]. Moreover, comparative analyses of pancreatic juice from patients with IPMN and concomitant PDAC have revealed distinct mutational profiles, underscoring the molecular heterogeneity of IPMN-associated neoplasia [19].

Experimental models have further clarified the cooperative nature of oncogenic pathways. Collet et al. showed that combined Kras and Lkb1 mutations synergistically induce IPMN-like lesions in murine ductal cells, providing in vivo evidence for the interplay between oncogenic drivers and tumor suppressor loss in IPMN initiation [20].

**Figure 1:**
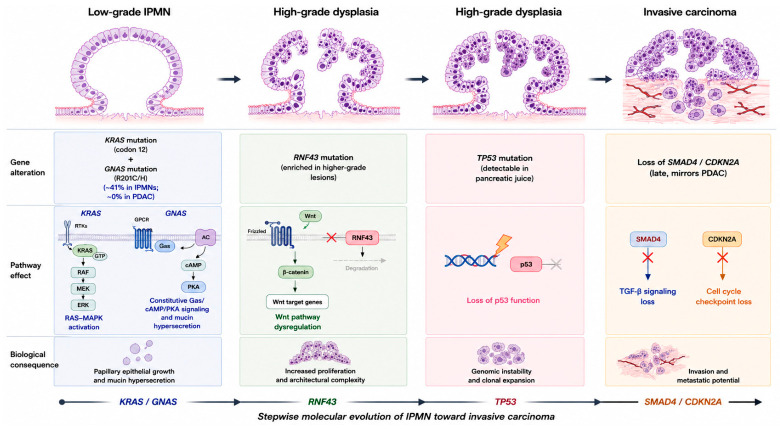
Molecular pathogenesis of IPMN: early drivers and progression. IPMN initiation is driven by activating mutations in KRAS (codon 12, RAS-MAPK activation) and GNAS (R201C/H, constitutive Gαs/cAMP/PKA signaling and mucin hypersecretion), the latter present in ~41% of IPMNs but essentially absent in conventional PDAC; co-occurrence of both mutations characterizes the gastric and intestinal subtypes. Progression to high-grade dysplasia and invasive carcinoma is driven by sequential acquisition of RNF43 mutations (Wnt pathway dysregulation, enriched in higher-grade lesions), TP53 mutations (detectable in pancreatic juice, a candidate surveillance biomarker), and late loss of SMAD4/CDKN2A, mirroring the molecular evolution of conventional PDAC.

The pathogenesis of IPMN is represented in Figure 1.

### 2.3. Subtype-Specific Molecular Pathways

A major conceptual advance in recent years has been the recognition that the four epithelial subtypes of IPMN—gastric, intestinal, pancreatobiliary, and oncocytic—represent biologically distinct molecular entities, rather than mere morphological variants. In a comprehensive analysis of 103 IPMN lesions from 43 patients, Kobayashi et al. demonstrated that each subtype exhibits unique mutational signatures and developmental trajectories [21]. This finding overturns the longstanding “myth” of IPMN as a single disease with variable histology and establishes a new “reality”: IPMN subtypes follow distinct molecular pathways with divergent clinical implications.

### 2.4. Pathways Linking IPMN and PDAC: Clonal Evolution and Field Effect

The relationship between IPMN and pancreatic ductal adenocarcinoma (PDAC) is increasingly recognized as more complex than a simple linear progression from precursor lesion to invasive cancer. Advances in molecular profiling have identified multiple evolutionary trajectories underlying the development of PDAC in patients with IPMN. In some cases, PDAC arises directly from the precursor lesion through a process of stepwise clonal evolution, retaining the major driver mutations acquired during IPMN progression. Alternatively, both lesions may originate from a common ancestral clone and subsequently diverge through the acquisition of distinct genetic alterations, resulting in parallel evolutionary pathways. A third scenario involves the development of PDAC as an independent neoplasm within the same pancreas, without any detectable clonal relationship to the concomitant IPMN. Collectively, these models highlight the biological heterogeneity of pancreatic carcinogenesis and underscore the diverse mechanisms through which invasive cancer may emerge in patients harboring IPMNs [22].

The recognition of these distinct evolutionary patterns has important clinical implications. Rather than representing an isolated precursor lesion, IPMN is increasingly viewed as a manifestation of a broader pancreatic field defect, in which the entire gland exhibits an increased susceptibility to neoplastic transformation. This concept provides a biological explanation for the persistent risk of PDAC observed even after complete surgical resection of an IPMN and supports the recommendation for continuous surveillance in selected patients [2]. Additional evidence for this model has been provided by Pflüger et al., who demonstrated that some post-resection recurrences originate from clonally unrelated lineages, suggesting the emergence of new neoplastic events rather than true local relapse [23]. Similarly, studies of uncommon histological variants have revealed highly complex evolutionary patterns. For example, Matsuzaka et al. reconstructed the molecular evolution of an adenosquamous carcinoma arising in association with IPMN, illustrating the branching and heterogeneous nature of tumor progression within the pancreatic epithelium [24].

## 3. Epidemiology and Incidence

### 3.1. Incidence Trends and the Role of Detection Bias

Over the past two decades, the reported incidence of IPMN has increased dramatically. This rise is widely interpreted as a consequence of the exponential growth in the use of high-resolution cross-sectional imaging—CT and MRI/MRCP—rather than a true biological surge in disease prevalence. Pancreatic cystic lesions are now identified in up to 45% of individuals undergoing abdominal imaging, and among these, 24–82% represent IPMNs, depending on the population studied and the imaging modality employed [1,25]. This remarkably broad range underscores one of the central epidemiological challenges in the field: distinguishing clinically meaningful IPMNs from incidental, indolent cysts that are unlikely to progress to malignancy.

Demographic factors further contribute to the rising incidence. Aronsson et al. (2017) highlighted that IPMN prevalence increases with age, making the aging population in developed countries a key driver of the observed epidemiological trends [25]. The disease exhibits a slight male predominance and typically presents in the sixth to seventh decades of life [5]. As detection rates rise, so too does the economic burden associated with IPMN, given the need for serial imaging; endoscopic evaluations; and, in selected cases, complex pancreatic surgery. These demands place increasing pressure on healthcare systems worldwide [7,25].

### 3.2. Trends in Malignant IPMN

Population-level analyses have provided important insights into the epidemiology of malignant IPMN. A Surveillance, Epidemiology, and End Results (SEER) database study spanning 1990–2010 documented a progressive increase in the identification of malignant IPMN in the United States, along with detailed survival trends and prognostic determinants [3]. The findings reinforced the critical importance of early-stage detection, as outcomes were strongly influenced by stage at diagnosis and the feasibility of surgical resection.

Beyond pancreatic malignancy, IPMN appears to confer a broader oncologic risk. A separate SEER-based study demonstrated an increased incidence of second primary cancers following a diagnosis of malignant IPMN, suggesting that affected patients warrant heightened vigilance not only for pancreatic neoplasia but also for extrapancreatic malignancies [26]. This observation aligns with the emerging concept of IPMN as part of a systemic or field-wide predisposition to neoplastic transformation.

### 3.3. Geographic Variation and the Evolving Research Landscape

Geographic patterns in IPMN research and incidence offer additional layers of complexity. Bibliometric analyses indicate that Japan has historically led global IPMN research, reflecting both a high prevalence of pancreatic cystic lesions in East Asian populations and a longstanding tradition of advanced pancreatic surgery and surveillance programs. South Korea and the United States have also emerged as major contributors to the scientific literature [6]. Whether these geographic differences reflect true biological variation or simply differences in diagnostic intensity, clinical awareness, and healthcare infrastructure remains unresolved.

What is clear, however, is that the global research agenda has shifted markedly over time. Early studies were dominated by descriptive pathology and disease characterization, whereas contemporary research increasingly focuses on management strategies, prognostic modeling, and biomarker development. Keywords such as “management,” “prognosis,” and “risk factors” now define the most active research fronts [6], mirroring the clinical need for more precise risk stratification and individualized surveillance algorithms.

## 4. Clinical Presentation: Signs and Symptoms

### 4.1. The Asymptomatic Majority

Paradoxically, the most common clinical presentation of IPMN is the complete absence of symptoms. The vast majority of lesions are detected incidentally during imaging performed for unrelated reasons—a trend that has accelerated with the widespread use of abdominal CT and MRI/MRCP [1,25]. This “incidentaloma” phenomenon introduces a substantial selection bias into the literature: asymptomatic lesions identified early tend to exhibit a more indolent biological behavior, thereby skewing natural history data toward more favorable outcomes.

### 4.2. Symptomatic Presentation

Although most IPMNs are detected incidentally, a subset of patients develop symptoms as a consequence of progressive ductal obstruction, mucin hypersecretion, or extensive involvement of the pancreatic parenchyma. Clinical manifestations are generally nonspecific and may reflect the diverse ways in which these lesions alter pancreatic structure and function.

Abdominal pain is the most frequently reported symptom and is typically described as vague, intermittent, and localized to the epigastrium. It is thought to result from increased intraductal pressure caused by mucin accumulation, ductal dilation, or local mass effect. Acute pancreatitis represents another common presentation, occurring in up to 20–30% of symptomatic patients. In these cases, recurrent episodes are usually triggered by intermittent obstruction of the main pancreatic duct or its side branches by mucin plugs, a mechanism that may closely resemble gallstone-related or idiopathic recurrent pancreatitis [5].

Longstanding ductal obstruction, particularly in MD- and mixed-type IPMNs, may eventually result in pancreatic exocrine and endocrine insufficiency [2,10]. Because these functional consequences represent long-term complications of disease progression and treatment, they are discussed in detail in Section 8.4.

In more advanced disease, involvement of adjacent structures may give rise to additional clinical findings. Obstructive jaundice is typically observed in patients with large main-duct lesions located in the pancreatic head and results from compression or invasion of the intrapancreatic bile duct. Because of its strong association with advanced neoplasia, jaundice is considered a high-risk stigma for malignancy in all major management guidelines. A palpable abdominal mass is uncommon and generally indicates a substantial tumor burden or advanced-stage disease.

Overall, while these manifestations may raise clinical suspicion for IPMN, none are pathognomonic, and their presence should be interpreted in conjunction with radiological, endoscopic, and pathological findings.

### 4.3. Symptoms as Predictors of Malignancy

A persistent “myth” in IPMN management is the assumption that asymptomatic lesions are uniformly benign. While symptomatic IPMNs indeed carry a higher likelihood of harboring high-grade dysplasia or invasive carcinoma, a substantial proportion of malignant IPMNs are asymptomatic at diagnosis [5]. Conversely, symptoms such as pancreatitis may arise from entirely benign lesions. The clinical challenge lies in contextualizing symptoms within a broader risk framework. The 2017 revised International Association of Pancreatology (IAP) guidelines acknowledge this nuance by incorporating new-onset or worsening diabetes among the “worrisome features” and obstructive jaundice among the “high-risk stigmata” [1,27]. These additions reflect the incremental—but imperfect—predictive value of clinical presentation when integrated with imaging, cyst morphology, and molecular markers. In addition, acute pancreatitis per se does not appear to increase the risk of malignant transformation in BD-IPMN lacking worrisome features or high-risk stigmata [28].

## 5. Diagnosis

### 5.1. Imaging Modalities

The diagnostic evaluation of IPMN relies on a multimodal strategy that integrates cross-sectional imaging, endoscopic assessment, and cyst fluid analysis [9,29]. Each modality contributes complementary information, and together they form the backbone of contemporary diagnostic algorithms.

**Figure 2:**
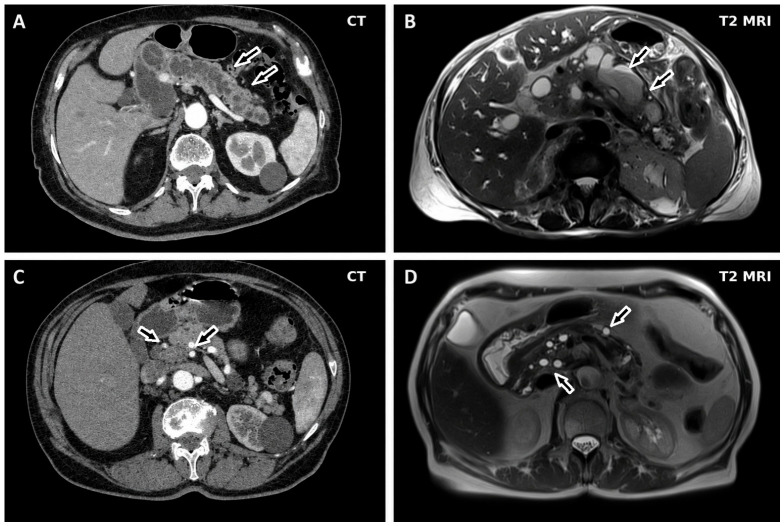
Representative cross-sectional imaging findings of main-duct and branch-duct intraductal papillary mucinous neoplasms (IPMNs). (**A**,**B**) Contrast-enhanced computed tomography (CT) (**A**) and axial T2-weighted magnetic resonance imaging (MRI) (**B**) from the same patient with main-duct IPMN (MD-IPMN), demonstrating diffuse dilatation of the main pancreatic duct (indicated by white arrows). (**C**,**D**) Contrast-enhanced CT (**C**) and axial T2-weighted MRI (**D**) from a patient with branch-duct IPMN (BD-IPMN), showing multiple fluid-signal cystic lesions arising from the branch ducts and communicating with the main pancreatic duct (indicated by white arrows).

Contrast-enhanced multidetector CT with a dedicated pancreatic protocol remains the most widely used initial imaging modality (Figure 2A,C). CT provides excellent spatial resolution for characterizing cyst morphology, assessing main pancreatic duct (MPD) diameter, identifying mural nodules, detecting calcifications, and evaluating parenchymal atrophy. It is also indispensable for staging suspected invasive IPMN and for assessing vascular involvement.

Magnetic Resonance Imaging (MRI) with Magnetic Resonance Cholangiopancreatography (MRCP) is generally considered the preferred modality for IPMN characterization and long-term surveillance (Figure 2B,D). Its advantages include superior soft-tissue contrast, absence of ionizing radiation—an essential consideration for lifelong follow-up—and unparalleled ability to delineate ductal communication, a hallmark feature distinguishing IPMN from other cystic neoplasms [29]. Min et al. (2021) evaluated the diagnostic performance of the 2017 international consensus guidelines utilizing CT and MRI, demonstrating that MRI/MRCP outperformed CT in detecting mural nodules and MPD dilation, two key features associated with malignancy [30].

Endoscopic Ultrasound (EUS) offers the highest-resolution imaging of pancreatic cystic lesions and enables fine needle aspiration (FNA) for cyst fluid analysis. It is recommended as a second-line modality when cross-sectional imaging reveals worrisome features or when further characterization is required. EUS allows detailed evaluation of mural nodules—including with contrast-enhanced techniques—cyst wall thickness, and internal architecture.

More recently, EUS-guided fine-needle biopsy (FNB) and through-the-needle microforceps biopsy have emerged as complementary diagnostic tools [31]. FNB may provide histological architecture in selected solid components, whereas microforceps biopsy enables direct sampling of the cyst wall, potentially improving diagnostic accuracy and dysplasia grading compared with cytology alone. However, both techniques remain reserved for selected cases because of procedural complexity and the need for further validation.

### 5.2. Cyst Fluid Analysis

Endoscopic ultrasound-guided fine-needle aspiration (EUS-FNA) allows the acquisition of cyst fluid for biochemical, cytological, and molecular analyses, providing important complementary information when imaging findings are inconclusive. Although no single cyst fluid marker can reliably establish the diagnosis or predict malignant transformation, the combined interpretation of multiple parameters may significantly improve diagnostic accuracy.

Among biochemical markers, carcinoembryonic antigen (CEA) remains the most widely used indicator of mucinous differentiation. Elevated CEA levels, particularly above the commonly adopted threshold of 192 ng/mL, support a mucinous origin of the cyst but do not reliably distinguish IPMNs from mucinous cystic neoplasms (MCNs) nor benign from malignant mucinous lesions. Consequently, its primary clinical value lies in confirming the mucinous nature of a pancreatic cyst rather than assessing its malignant potential.

Amylase concentration provides complementary information regarding communication with the pancreatic ductal system. High amylase levels generally favor the diagnosis of IPMN, reflecting direct ductal communication, whereas low levels are more consistent with non-communicating cystic lesions such as serous cystadenomas. Cytological examination, although highly specific when atypical or malignant cells are identified, is limited by the frequently paucicellular nature of pancreatic cyst aspirates, resulting in a sensitivity of approximately 50%.

More recently, molecular analysis of cyst fluid has emerged as one of the most promising advances in the diagnostic evaluation of pancreatic cystic lesions. The detection of characteristic mutations, particularly involving KRAS and GNAS, as well as the application of next-generation sequencing (NGS) panels, has improved the ability to characterize cyst subtype and assess the risk of progression. Integrated molecular pathology (IMP) models further combine molecular findings with clinical and radiological variables to refine risk stratification. In this context, Simpson et al. demonstrated that simplifying the IMP algorithm by excluding most clinical variables, with the exception of main pancreatic duct diameter, improved the discrimination of invasive or malignant disease [32]. In parallel, the search for novel biomarkers continues to evolve, with preliminary studies suggesting that cyst fluid proteins such as HMGA2 may help differentiate low-grade from high-grade dysplasia and thereby enhance preoperative risk assessment [14,32,33].

Overall, molecular profiling is progressively reshaping the diagnostic approach to IPMNs and may play an increasingly important role in personalized risk stratification as these technologies become more widely available and clinically validated.

### 5.3. Endoscopic Findings

Classic endoscopic findings of main-duct and mixed-type IPMN include the “fish-mouth” or “fish-eye” papilla, characterized by a patulous ampulla of Vater with mucin extrusion. Although pathognomonic, this sign is infrequently observed.

Endoscopic retrograde cholangiopancreatography (ERCP), once central to diagnosis, has largely been replaced by MRCP due to its invasiveness and risk profile, though it retains a role in selected therapeutic interventions.

Peroral pancreatoscopy (POP) has emerged as a valuable adjunct for direct visualization of the pancreatic ductal system. A systematic review and meta-analysis by de Jong et al. (2022) encompassing 25 studies demonstrated high cannulation and visualization rates, with consistently strong diagnostic accuracy [34]. Reported visual features include fish-egg-like lesions, hypervascularity, and granular mucosa. Importantly, POP altered surgical management in 13–62% of cases. However, its adverse event rate of 12%—primarily post-ERCP pancreatitis—remains a significant limitation [34].

### 5.4. Predictive Models and Risk Stratification

Recent research has increasingly focused on developing quantitative, imaging-based predictive models to refine malignancy risk assessment. Park et al. (2024) proposed and validated a multiparametric imaging model capable of estimating malignancy risk in IPMN, reflecting the broader shift toward data-driven risk stratification [35]. These models aim to transcend the binary “high-risk stigmata” versus “worrisome features” framework of current guidelines. Hasegawa et al. (2024) further advanced this field by integrating the age-adjusted Charlson comorbidity index with imaging features such as solid components, thereby incorporating patient fitness into surgical decision-making [36]. This approach reflects a growing recognition that optimal management requires balancing oncologic risk with individualized assessment of operative suitability.

## 6. Classification, Macroscopic Characteristics, and Pathological Features

### 6.1. Anatomical Classification

**Figure 3:**
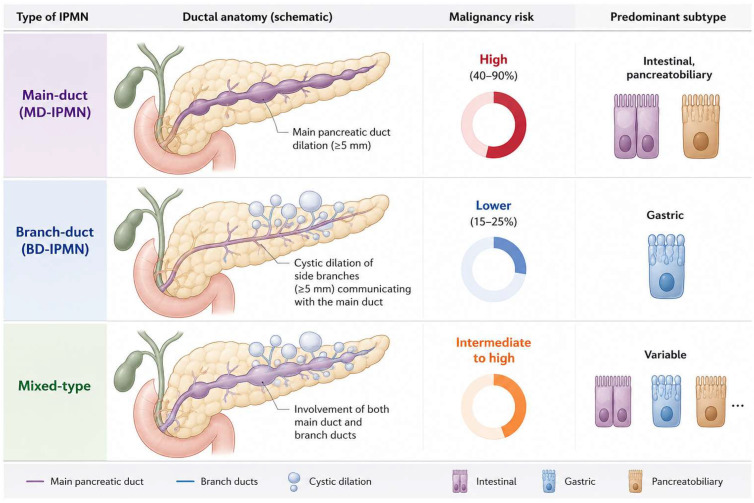
Anatomical classification of IPMNs and their association with malignant risk and epithelial subtype. Representative schematics of main-duct (MD-IPMN), branch-duct (BD-IPMN), and mixed-type IPMNs. MD-IPMNs are characterized by dilatation of the main pancreatic duct and have the highest risk of malignant progression (40–90%), predominantly showing intestinal or pancreatobiliary differentiation. BD-IPMNs arise from cystic branch-duct dilatation and have a lower risk of malignancy (15–25%), most commonly exhibiting a gastric phenotype. Mixed-type IPMNs involve both the main duct and branch ducts and display intermediate-to-high malignant potential with variable histological differentiation. Colors indicate the main pancreatic duct (purple), branch ducts and cystic lesions (blue), and representative epithelial subtypes.

IPMNs are classified based on the ductal segment involved in Main-duct (MD-IPMN), Branch-duct (BD-IPMN), and mixed type. Each one of this subtype presents peculiar characteristics and malignant risk (Figure 3).

This classification, first formalized in the Sendai guidelines (2006) and refined in the Fukuoka (2012) and revised 2017 guidelines, remains the cornerstone of clinical risk stratification [2,9]. The malignant risk of main-duct and mixed-type IPMN ranges broadly from approximately 40% to 90%, while the malignancy risk for BD-IPMN is more variable but considered to be substantially lower [1].

### 6.2. Histological Subtypes

According to the current WHO classification, IPMNs can be categorized into four major epithelial subtypes based on their morphological, immunohistochemical, and molecular characteristics. These subtypes are increasingly recognized as biologically distinct entities rather than merely descriptive histological variants, as accumulating evidence suggests that they differ in their molecular pathogenesis, propensity for malignant transformation, and clinical outcomes.

The gastric subtype is the most prevalent form, accounting for approximately 50–70% of all IPMNs. Histologically, it is characterized by pyloric gland-like epithelium with strong MUC5AC expression and is most commonly identified in branch-duct lesions. Gastric-type IPMNs typically exhibit low-grade dysplasia and are frequently associated with KRAS mutations. When invasive transformation occurs, the resulting carcinoma is usually of the tubular adenocarcinoma type. Overall, this subtype is associated with the lowest malignant potential and the most favorable prognosis among conventional IPMNs.

In contrast, the intestinal subtype displays a villous architecture resembling that of colonic adenomas and is characterized by expression of MUC2 and CDX2. It is predominantly encountered in main-duct IPMNs and commonly harbors both KRAS and GNAS mutations. A distinctive feature of this subtype is its tendency to progress to colloid carcinoma when invasion develops. Because colloid carcinoma generally exhibits a more indolent biological behavior than conventional tubular pancreatic ductal adenocarcinoma, patients with invasive intestinal-type IPMN often experience more favorable outcomes.

The pancreatobiliary subtype represents the least common but clinically most aggressive conventional IPMN variant. It is characterized by complex branching papillae supported by delicate fibrovascular cores and typically expresses MUC1. Compared with the gastric and intestinal subtypes, pancreatobiliary IPMNs are more frequently associated with high-grade dysplasia and invasive tubular adenocarcinoma, translating into a less favorable prognosis.

A distinct category is represented by the oncocytic subtype, which was reclassified in the 2019 WHO classification as intraductal oncocytic papillary neoplasm (IOPN). Histologically, IOPN is characterized by elaborate arborizing papillae lined by oncocytic cells containing abundant eosinophilic, mitochondria-rich cytoplasm. Unlike conventional IPMNs, these neoplasms typically lack the canonical KRAS and GNAS mutations and instead exhibit a unique molecular landscape, supporting their recognition as a separate pathological entity. Despite their often complex morphology, IOPNs are generally associated with favorable long-term outcomes.

The clinical relevance of histological subclassification extends beyond pathological diagnosis. Del Chiaro and Verbeke emphasized that several macroscopic and microscopic features correlate with the risk of malignancy and should therefore be systematically reported [37]. Furthermore, molecular studies have provided strong evidence that the different epithelial subtypes arise through distinct developmental pathways, reinforcing the concept that they represent separate biological entities rather than stages of a single disease spectrum [21]. Consistent with this view, Chang et al. demonstrated significant associations between KRAS mutational status, histological subtype, and clinicopathological characteristics, further highlighting the interplay between morphology and molecular evolution in IPMN progression [18].

### 6.3. Macroscopic Features

Macroscopically, IPMNs are characterized by intraductal epithelial proliferations that produce varying degrees of cystic dilation of the pancreatic ductal system. The gross appearance of these lesions largely reflects their anatomical distribution and biological behavior, providing important clues for diagnosis and risk stratification.

One of the most distinctive findings is the extrusion of mucin through the ampulla of Vater, resulting in the characteristic “fish-eye” or “fish-mouth” appearance. Although considered highly specific for IPMN, this finding is encountered in only a minority of patients. More commonly, the macroscopic appearance is determined by the pattern of ductal involvement. Branch-duct IPMNs typically present as unilocular or multilocular cystic lesions with a grape-like configuration, whereas main-duct IPMNs are characterized by diffuse or segmental dilation of the main pancreatic duct. Lesion size also carries important clinical implications, as cysts measuring 3 cm or larger are considered a worrisome feature in current management guidelines.

Particular attention should be paid to the presence of mural nodules, which represent one of the strongest macroscopic predictors of advanced neoplasia and invasive disease. The risk is especially increased when these nodules demonstrate contrast enhancement and measure at least 5 mm in diameter. Accurate differentiation between true mural nodules and intraluminal mucin aggregates, commonly referred to as mucin balls, is therefore essential during radiological and endoscopic evaluation, as the latter lack vascularization and do not carry the same prognostic significance.

The cystic spaces are typically filled with thick, viscous mucin, reflecting the secretory activity of the neoplastic epithelium. This feature may also be appreciated during EUS-guided aspiration, where the presence of highly viscous fluid producing a positive “string sign” supports the diagnosis of a mucinous cystic lesion. Another key morphological parameter is the diameter of the main pancreatic duct. Current guidelines recognize a ductal diameter of 5 mm or greater as a worrisome feature, whereas dilation of 10 mm or more constitutes a high-risk stigma strongly associated with malignancy in main-duct and mixed-type IPMNs.

Finally, chronic ductal obstruction may induce secondary changes in the surrounding pancreatic parenchyma. Upstream glandular atrophy is frequently observed in longstanding disease and reflects the progressive impact of ductal dilation and impaired pancreatic drainage on the adjacent exocrine tissue. Together, these macroscopic features not only facilitate diagnosis but also provide important prognostic information that contributes to clinical decision-making and surgical risk assessment.

### 6.4. Differential Diagnosis

The differential diagnosis of IPMN encompasses several pancreatic cystic and solid lesions (Table 1). Chelliah et al. (2016) provided a systematic overview of intraductal tubulopapillary neoplasms, highlighting the diagnostic confusion that can arise between these rare entities and conventional IPMNs and emphasizing the importance of precise categorization for prognostication [4]. The distinction between IPMN and MCN has been particularly refined by the recognition that ductal communication (demonstrated by MRCP) is the single most reliable discriminator between these two entities [9].

## 7. Surveillance and Follow-Up

### 7.1. Guideline-Based Risk Stratification

In the absence of definitive indications for surgery, the management of IPMNs relies on risk stratification strategies aimed at identifying lesions with a higher likelihood of harboring high-grade dysplasia or invasive carcinoma while minimizing unnecessary pancreatic resections. Among the available frameworks, the 2017 revised International Association of Pancreatology (IAP) guidelines remain the most widely adopted and classify IPMNs according to the presence of either high-risk stigmata or worrisome features, each associated with distinct management recommendations.

High-risk stigmata identify patients who should generally be considered for surgical resection if medically fit. These include obstructive jaundice associated with a cystic lesion in the pancreatic head, the presence of an enhancing mural nodule measuring at least 5 mm, marked dilation of the main pancreatic duct (≥10 mm), and cytological evidence of malignancy or high-grade dysplasia. The presence of any of these findings is strongly associated with advanced neoplasia and substantially increases the probability of invasive disease.

In contrast, worrisome features indicate an intermediate level of risk and warrant further evaluation, most commonly by endoscopic ultrasound. These features include cyst diameter of at least 3 cm, small enhancing mural nodules (<5 mm), thickened or enhancing cyst walls, main pancreatic duct dilation between 5 and 9 mm, abrupt changes in duct caliber associated with distal pancreatic atrophy, regional lymphadenopathy, elevated serum CA 19-9 levels, rapid cyst growth, and the development of new-onset or worsening diabetes mellitus. Although individually less predictive than high-risk stigmata, these findings may identify lesions requiring closer surveillance or more detailed diagnostic assessment.

The clinical performance of this risk-based approach has been evaluated in several studies. Watanabe et al. demonstrated that the 2017 IAP guidelines provide reasonable sensitivity for the detection of malignant IPMNs; however, their specificity remains suboptimal, as a considerable proportion of surgically resected lesions ultimately prove to harbor only low-grade dysplasia [27]. This observation highlights one of the central challenges in contemporary IPMN management: achieving an appropriate balance between early identification of malignant transformation and avoidance of unnecessary pancreatic surgery, which continues to carry substantial morbidity despite advances in surgical techniques and perioperative care.

### 7.2. Surveillance Protocols: Controversies and Discrepancies

Despite broad agreement regarding the major risk factors for malignancy, substantial differences remain among international surveillance guidelines, reflecting the persistent uncertainty surrounding the natural history of IPMNs and the limited availability of high-quality prospective evidence. Consequently, surveillance recommendations vary considerably with respect to imaging intervals, duration of follow-up, and criteria for discontinuing monitoring.

The revised IAP/Fukuoka guidelines, originally introduced in 2012 and updated in 2017, advocate for continued surveillance for all branch-duct IPMNs lacking high-risk stigmata or worrisome features. Surveillance intervals are tailored according to cyst characteristics and generally range from 6 to 24 months, with long-term or lifelong follow-up frequently recommended. In contrast, the 2015 American Gastroenterological Association (AGA) guidelines proposed a more conservative strategy, suggesting that surveillance may be discontinued after five years of stability in the absence of concerning findings. This recommendation generated substantial debate, as subsequent studies documented the occurrence of malignant transformation beyond the five-year surveillance window. The 2018 European evidence-based guidelines adopted a more intermediate position, supporting continued monitoring while allowing longer surveillance intervals for selected low-risk lesions that remain stable over time. More recently, the 2024 Kyoto guidelines have incorporated emerging evidence and further refined risk stratification criteria, reflecting the ongoing evolution of the field [7,11,38].

Notwithstanding these differences, a common limitation of all currently available recommendations is that they remain largely consensus-driven. As highlighted by Levink et al., many surveillance strategies are based on expert opinion rather than robust prospective data, underscoring the need for more reliable predictors of malignant progression [7]. Furthermore, a significant proportion of IPMNs resected according to guideline-based criteria are ultimately found to harbor only low-grade dysplasia on final pathology. This observation challenges the assumption that current algorithms can accurately distinguish high-risk from low-risk disease and highlights the persistent risk of overtreatment. In this context, Mayhew et al. emphasized the importance of moving toward more individualized surveillance strategies that integrate not only cyst-related features but also patient-specific factors such as age, comorbidities, life expectancy, and operative risk [11]. Such an approach may ultimately provide a more balanced framework for decision-making than the exclusive reliance on morphological criteria.

An important area of ongoing debate concerns the duration of surveillance for small, stable BD-IPMNs. While the 2015 AGA guidelines proposed discontinuing surveillance after 5 years of stability, this recommendation has remained controversial because subsequent studies documented malignant progression beyond this time point. More recently, however, large international cohort studies have suggested that surveillance discontinuation may be reasonable in carefully selected patients. In particular, Marchegiani et al. demonstrated that in patients with presumed BD-IPMNs lacking worrisome features or high-risk stigmata, the risk of developing pancreatic cancer after 5 years of radiological stability becomes comparable to that of the age-matched general population when cyst size remains small and patient age is advanced [38]. Consistent with these findings, the 2024 Kyoto guidelines endorse a more individualized approach, allowing consideration of surveillance discontinuation in selected low-risk patients after prolonged stability, while emphasizing that this decision should account for cyst characteristics, life expectancy, comorbidities, and patient preferences [39].

### 7.3. Recommended Surveillance Protocol

**Figure 4:**
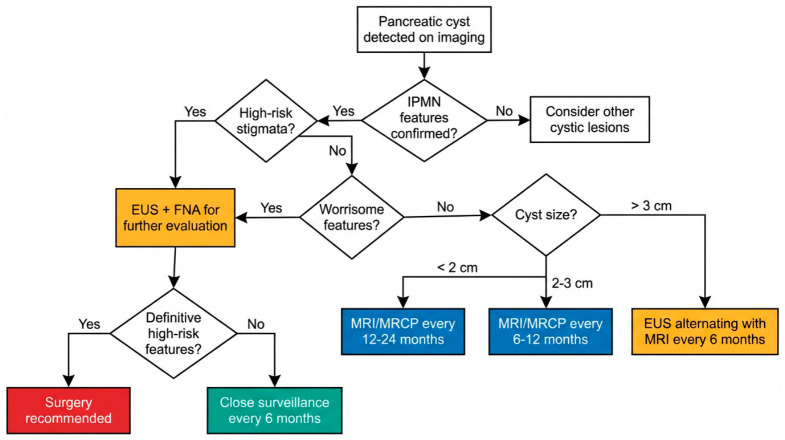
Simplified risk-stratified management algorithm for IPMNs. Schematic overview of the diagnostic work-up and surveillance strategy for patients with pancreatic cystic lesions suspected to be IPMNs. Clinical management is guided by the presence of high-risk stigmata, worrisome features, and cyst size. EUS-FNA is recommended for further evaluation of lesions with concerning features, whereas surveillance intervals are tailored according to cyst dimensions in low-risk lesions. Surgical resection is reserved for patients with definitive high-risk features suggestive of advanced neoplasia or invasive carcinoma.

From a practical perspective, effective surveillance should extend beyond the simple application of guideline thresholds and focus on the longitudinal assessment of disease behavior. Follow-up imaging should systematically evaluate cyst size, number, and anatomical distribution, together with the diameter of the main pancreatic duct, the presence of mural nodules or solid components, and associated parenchymal abnormalities such as pancreatic atrophy (Figure 4).

Equally important is the comparison with previous examinations, as temporal evolution often provides more clinically meaningful information than a single cross-sectional assessment. Progressive cyst enlargement, the appearance of new mural nodules, or increasing main pancreatic duct dilation may indicate biological progression and warrant closer monitoring or additional diagnostic evaluation. Conversely, prolonged stability generally supports a more conservative management strategy.

This dynamic approach reflects the current understanding that IPMNs represent a heterogeneous group of lesions with variable biological potential. Accordingly, surveillance should aim not only to identify established high-risk features but also to detect subtle changes over time that may signal the transition from indolent disease to clinically significant neoplasia.

### 7.4. Duration of Surveillance and the “Field Defect” Paradigm

The duration of surveillance remains one of the most debated and conceptually challenging aspects of IPMN management. Central to this debate is the recognition of IPMN as a “field defect”—a biological state in which the entire pancreas exhibits a predisposition to neoplastic transformation. This paradigm implies that new IPMNs may arise in previously unaffected regions of the gland and that pancreatic ductal adenocarcinoma (PDAC) may develop in the remnant pancreas even after complete resection of the index lesion [2]. This biological reality strongly supports the rationale for lifelong surveillance.

However, the benefits of indefinite monitoring must be balanced against cumulative costs, patient anxiety, and diminishing clinical returns in elderly or comorbid individuals. The challenge lies in determining when continued surveillance meaningfully contributes to patient outcomes and when it becomes disproportionately burdensome. Sekine et al. (2024) addressed this dilemma through a large multicenter study involving 1864 patients with IPMN, applying competing risk analysis to differentiate predictors of comorbidity-related mortality from those associated with pancreatic cancer-related mortality [40]. Their findings underscore a critical principle: surveillance and treatment decisions should be individualized, taking into account not only the biological behavior of the lesion but also the patient’s overall health status, comorbidities, and life expectancy. This patient-centered approach marks a shift away from rigid, guideline-driven algorithms toward a more nuanced framework that integrates oncologic risk with personalized assessment of benefit. As the field evolves, refining surveillance duration will require prospective data that better capture long-term outcomes across diverse patient populations.

## 8. Long-Term Complications

### 8.1. Malignant Transformation

The most clinically significant long-term complication of IPMN is progression to invasive carcinoma. However, the risk of malignant transformation is highly heterogeneous and largely depends on the pattern of ductal involvement. Main-duct IPMNs carry the highest risk, with reported malignancy rates ranging from 40% to 90% in surgical series. These estimates should be interpreted with caution, as they are derived predominantly from resected cohorts and are therefore influenced by selection bias toward higher-risk lesions. In contrast, branch-duct IPMNs generally exhibit a substantially lower risk of malignant progression, estimated at approximately 15–25%. Mixed-type IPMNs occupy an intermediate-to-high-risk position and are generally considered biologically similar to main-duct lesions with regard to their malignant potential [1,11,25].

Although the association between IPMN and invasive carcinoma is well established, the natural history of malignant transformation remains incompletely understood. In particular, the timing of progression and the factors determining why some lesions remain indolent for decades whereas others rapidly evolve toward invasive cancer continue to represent major unresolved questions. As highlighted by Ma et al., the biological mechanisms governing the transition from non-invasive neoplasia to invasive carcinoma remain only partially elucidated, constituting one of the most important knowledge gaps in the field [29].

Molecular studies have identified a series of genetic alterations associated with advanced neoplastic progression, including TP53 and SMAD4 inactivation, which are typically regarded as late events in the adenoma–carcinoma sequence of IPMN evolution [10,17]. Nevertheless, despite substantial advances in understanding the molecular landscape of these lesions, these discoveries have not yet translated into clinically validated predictive models capable of accurately identifying which patients will ultimately develop invasive disease. Consequently, the prevention of malignant transformation remains heavily reliant on imaging-based surveillance and risk stratification strategies rather than on molecularly guided decision-making.

A recent large multicenter study involving 3844 BD-IPMN patients demonstrated that the risk of developing pancreatic malignancy without worrisome features or high risk stigmata after 5 years of surveillance is comparable to that of the general population depending on cyst size and patient age. In those patients, surveillance discontinuation could be justified after 5 years of stability in patients older than 75 years with cysts <30 mm, and in patients 65 years or older who have cysts ≤15 mm [38].

### 8.2. Recurrence After Resection

Although surgical resection represents the definitive treatment for selected IPMNs, it does not eliminate the risk of subsequent neoplastic progression within the remnant pancreas. Recurrence therefore remains a major long-term concern and provides further support for the concept of IPMN as a manifestation of a diffuse pancreatic field defect rather than a purely localized lesion.

Evidence from large multicenter cohorts has demonstrated that clinically relevant recurrences may occur many years after apparently curative surgery. In a study including 1074 patients who underwent resection for IPMN, Hirono et al. reported an overall recurrence rate of 14.4%, with more than one-third of high-risk lesions arising in the remnant pancreas being diagnosed beyond five years after surgery [41]. These findings challenge the notion that postoperative risk substantially declines over time and provide a strong rationale for prolonged, and often lifelong, surveillance following resection.

Importantly, the same study showed that patients undergoing repeat surgery for metachronous high-risk lesions achieved significantly better survival than those who were managed non-operatively, highlighting the potential clinical benefit of early detection through structured postoperative follow-up (*p* = 0.04) [41]. Several factors were independently associated with the development of metachronous high-risk lesions, including symptomatic presentation at diagnosis, body or tail location of the primary lesion, marked main pancreatic duct dilation (≥10 mm), and the presence of high-grade dysplasia or invasive carcinoma in the resected specimen.

The clinical relevance of early intervention was further supported by Djoumi et al., who reported comparable long-term survival in patients undergoing pre-emptive resection of non-invasive IPMNs and those treated for early-stage invasive disease [42]. These findings suggest that timely identification and treatment of high-risk lesions may mitigate the adverse prognostic impact of malignant transformation.

Recent molecular studies have provided additional insight into the biological basis of postoperative recurrence. Pflüger et al. demonstrated that recurrent lesions may originate from clonally distinct cellular populations rather than from residual disease left behind at surgery [23]. This observation reinforces the field-defect hypothesis supporting the concept discussed above that postoperative lesions may arise independently rather than from residual disease. Consequently, postoperative surveillance should not be considered merely a strategy for detecting local relapse but rather an essential component of long-term disease management aimed at identifying newly emerging high-risk lesions.

Invasive IPMN exhibits relapse patterns that differ from those of conventional PDAC. Capretti et al. (2022) showed that invasive IPMN tends to relapse later and more frequently in the lungs, suggesting a biologically distinct metastatic behavior [43]. These findings support the concept that IPMN-derived carcinomas represent a biologically unique entity, with implications for postoperative surveillance strategies and potentially for systemic therapy selection.

### 8.3. Peritoneal Metastases

Peritoneal metastasis is a rare but recognized complication following resection of malignant IPMN. Snow et al. (2023) reviewed institutional and published data, noting that although uncommon, peritoneal metastases carry a poor prognosis, and their true prevalence and risk factors remain poorly defined due to limited case numbers [44]. Mechanistically, peritoneal dissemination is thought to result from mucin spillage into the peritoneal cavity, either through spontaneous rupture of a main-duct IPMN or intraoperative leakage.

### 8.4. Pancreatic Exocrine and Endocrine Insufficiency

Beyond the risk of malignant transformation, IPMNs may lead to significant long-term functional impairment of the pancreas. Longstanding ductal obstruction is primarily observed in MD-IPMN and mixed-type IPMN, where diffuse involvement of the main pancreatic duct may lead to progressive pancreatic atrophy, exocrine insufficiency, and pancreatogenic diabetes. Progressive ductal obstruction caused by mucin accumulation and ductal dilation can induce chronic injury to the surrounding parenchyma, ultimately resulting in both exocrine and endocrine insufficiency. These complications may arise as a direct consequence of the disease itself or be exacerbated by surgical treatment.

Exocrine pancreatic insufficiency develops as a result of progressive loss of acinar tissue and impaired pancreatic secretion. Clinically, affected patients may present with maldigestion, steatorrhea, weight loss, and deficiencies of fat-soluble vitamins, including vitamins A, D, E, and K. If left untreated, these alterations can contribute to progressive malnutrition and reduced quality of life. Consequently, pancreatic enzyme replacement therapy (PERT) often represents a key component of long-term management, particularly in patients with extensive ductal involvement or after pancreatic resection [2].

Endocrine dysfunction may also occur due to the gradual destruction of islet-rich pancreatic tissue. This can manifest as new-onset diabetes mellitus or worsening glycemic control in patients with pre-existing diabetes, a condition often classified as pancreatogenic (type 3c) diabetes [10]. In addition to disease-related parenchymal loss, endocrine insufficiency may be precipitated or aggravated by pancreatic surgery, especially following extensive resections such as pancreatoduodenectomy or total pancreatectomy.

Surgical treatment itself represents an additional source of long-term morbidity. While resection remains the cornerstone of management for high-risk lesions, postoperative complications including pancreatic fistula, anastomotic dysfunction, and the metabolic consequences of reduced pancreatic reserve may significantly affect long-term outcomes. Taken together, these considerations highlight that the burden of IPMN extends beyond oncological risk alone and includes substantial functional consequences that should be incorporated into both surveillance strategies and therapeutic decision-making.

## 9. Clinical Syndromes, Associated Conditions, and Management Challenges

### 9.1. Associated Conditions and Risk Modifiers

#### 9.1.1. Extrapancreatic Malignancies

An intriguing and still debated aspect of IPMN biology is its reported association with an increased incidence of extrapancreatic malignancies. Several observational studies have suggested that patients with IPMN may be at higher risk of developing second primary cancers, raising the possibility that IPMN represents not only a localized pancreatic neoplasm but also a marker of broader cancer susceptibility. Supporting this hypothesis, Huang et al. identified an increased incidence of malignancies involving the colorectum, stomach, kidney, and thyroid among patients with malignant IPMN in a population-based SEER analysis [26].

The mechanisms underlying this association remain incompletely understood. Several explanations have been proposed, including shared genetic susceptibility factors, such as defects in DNA repair pathways or germline mutations involving BRCA2 and related genes, as well as surveillance bias resulting from the intensive imaging follow-up commonly performed in these patients. Another possibility is that IPMN may represent one manifestation of a broader cancer-prone phenotype driven by molecular alterations that extend beyond the pancreas. Although definitive evidence remains lacking, clarification of this relationship could have important clinical implications, potentially supporting more individualized cancer screening strategies in selected high-risk patients.

#### 9.1.2. Chronic Pancreatitis

The chronic obstruction of the pancreatic ductal system caused by mucin-producing neoplastic epithelium may result in recurrent episodes of acute pancreatitis, which over time can evolve into chronic pancreatitis. This progression contributes to a spectrum of complications including chronic abdominal pain, exocrine pancreatic insufficiency, endocrine dysfunction, and impaired quality of life. From a diagnostic perspective, the coexistence of chronic pancreatitis and IPMN presents additional challenges, as inflammatory changes may obscure mural nodules, alter ductal morphology, and reduce the accuracy of radiological surveillance. Consequently, distinguishing disease progression from inflammation-related abnormalities often requires a multimodal approach incorporating imaging, endoscopy, and longitudinal follow-up.

#### 9.1.3. Familial Pancreatic Cancer Syndromes

Accumulating evidence suggests that IPMN may occur more frequently in individuals with hereditary predisposition to pancreatic cancer. Associations have been reported with familial pancreatic cancer kindreds as well as several well-characterized hereditary cancer syndromes, including those related to BRCA2, PALB2, CDKN2A, STK11 (Peutz–Jeghers syndrome), and mismatch repair gene mutations associated with Lynch syndrome. These observations support the concept that inherited susceptibility may contribute not only to pancreatic ductal adenocarcinoma but also to the development of precursor lesions such as IPMN.

The biological relevance of genetic predisposition is further highlighted by the reported association between McCune–Albright syndrome and IPMN-associated pancreatic cancer, a relationship thought to be mediated by germline GNAS mosaicism [15]. In clinical practice, the presence of a strong family history of pancreatic cancer or a known hereditary cancer syndrome may justify more intensive surveillance strategies and a lower threshold for further diagnostic evaluation or surgical intervention.

#### 9.1.4. Metabolic and Vascular Associations

Emerging evidence has suggested possible links between IPMN and metabolic conditions such as obesity, metabolic syndrome, and type 2 diabetes mellitus. However, the interpretation of these associations remains challenging because these conditions are highly prevalent in the same aging population in which IPMNs are commonly diagnosed. It therefore remains unclear whether these relationships reflect causal biological mechanisms, including chronic hyperinsulinemia and growth factor-mediated stimulation of ductal epithelium, or simply the coexistence of shared epidemiological risk factors.

At present, available data are insufficient to establish metabolic factors as independent predictors of IPMN progression or malignant transformation. Dedicated prospective studies are needed before these associations can be incorporated into risk stratification models or surveillance algorithms.

### 9.2. Challenges and Burden of Management

#### 9.2.1. Surgical Morbidity and Mortality

Although surgical resection remains the cornerstone of treatment for high-risk IPMNs, pancreatic surgery continues to carry substantial perioperative and long-term morbidity. Procedures such as pancreatoduodenectomy, distal pancreatectomy, and total pancreatectomy are associated with significant complication rates, including pancreatic fistula, delayed gastric emptying, postoperative hemorrhage, biliary complications, and intra-abdominal infections. In a 13-year single-center experience, Marsoner et al. confirmed the considerable morbidity associated with IPMN resection, particularly among patients with invasive disease [45].

While perioperative mortality has progressively declined and now approaches 1–3% in experienced high-volume centers, the risks associated with surgery remain clinically relevant, especially when balanced against the often indolent natural history of low-risk branch-duct IPMNs [8]. These considerations underscore the importance of accurate preoperative risk stratification. Recent approaches have increasingly incorporated patient-related factors into surgical decision-making. For example, Hasegawa et al. proposed criteria integrating the age-adjusted Charlson Comorbidity Index with radiological features such as solid components, aiming to identify patients for whom operative risks may outweigh potential oncological benefits [36]. Similarly, the Kyoto 2024 guidelines emphasize the importance of patient fitness, frailty, and life expectancy when considering surgical intervention [39].

#### 9.2.2. Surveillance-Related Burden

The benefits of long-term surveillance must be weighed against its economic and psychological consequences. Repeated MRI/MRCP examinations, endoscopic ultrasound procedures, specialist consultations, and prolonged follow-up generate substantial healthcare costs, particularly given the often lifelong nature of surveillance programs. Aronsson et al. demonstrated that surveillance is generally more cost-effective than immediate surgery for low-risk branch-duct IPMNs, although cumulative expenditures remain considerable over time [46].

Beyond financial considerations, surveillance may impose a significant psychological burden on patients. The awareness of harboring a potentially premalignant lesion, combined with the uncertainty associated with repeated imaging assessments, may generate chronic anxiety and reduced quality of life. This phenomenon has occasionally been described as the “Damocles syndrome,” reflecting the persistent perception of an impending health threat. Although relatively understudied, these psychological aspects deserve greater attention when designing patient-centered surveillance strategies.

#### 9.2.3. Competing Causes of Mortality

One of the most important contemporary challenges in IPMN management is the recognition that the risk of pancreatic cancer must be interpreted within the broader context of patient health status. Many individuals diagnosed with IPMN are elderly and frequently have multiple comorbid conditions that substantially influence long-term survival. Consequently, the probability of death from cardiovascular disease, other malignancies, or age-related illnesses may exceed the risk of progression to pancreatic cancer, particularly in patients with low-risk branch-duct lesions.

This concept has important implications for both surveillance and surgical decision-making. Using competing-risk analysis in a large multicenter cohort, Sekine et al. identified distinct predictors of pancreatic cancer-related and comorbidity-related mortality, demonstrating that these outcomes cannot be considered independently when designing follow-up strategies [40]. Their findings support a more individualized approach in which age, frailty, life expectancy, and comorbidity burden are integrated alongside traditional radiological risk factors. For an elderly patient with a small, stable branch-duct IPMN and significant medical comorbidities, the likelihood of dying from unrelated causes may greatly exceed the probability of IPMN-related mortality. In such circumstances, the benefits of intensive surveillance or prophylactic surgery become increasingly uncertain and should be carefully balanced against their potential risks and burdens.

Taken together, these observations highlight the need to move beyond a purely lesion-centered approach toward a more comprehensive model of IPMN management that integrates biological risk, patient characteristics, quality of life, and competing health priorities.

## 10. Therapeutic Approach: Current Practice and Future Perspectives

### 10.1. Surgical Resection: Indications and Techniques

Surgical resection remains the only potentially curative treatment for IPMNs harboring high-grade dysplasia or invasive carcinoma. Current recommendations are largely based on the anatomical classification (i.e., MD- vs. BD-IPMN), and presence of high-risk stigmata or worrisome features associated with concerning endoscopic findings, as defined by the revised 2017 International Association of Pancreatology (IAP) guidelines and further refined in the 2024 Kyoto consensus [1,39]. The extent of resection is determined by the anatomical distribution of disease, ranging from segmental pancreatectomy to more extensive procedures in patients with diffuse ductal involvement (Table 2).

Standard oncological resections remain the preferred approach for patients with MD-IPMN, mixed-type IPMN, or lesions harboring high-grade dysplasia or invasive carcinoma, as they ensure adequate lymphadenectomy and oncological clearance. Conversely, parenchyma-sparing procedures—including central pancreatectomy and enucleation—may be considered in carefully selected patients with low-risk, non-invasive lesions located in favorable anatomical positions, particularly younger individuals in whom preservation of endocrine and exocrine function is a priority [47]. Total pancreatectomy should be reserved for diffuse main-duct involvement, multifocal disease not amenable to segmental resection, or persistent high-grade dysplasia at the resection margin, balancing oncological benefit against the substantial metabolic consequences of complete pancreatic removal.

A fundamental objective of surgery is the achievement of oncologically adequate resection while preserving as much functional pancreatic parenchyma as possible. For this reason, intraoperative frozen-section analysis of the pancreatic margin is routinely recommended. The identification of high-grade dysplasia or invasive carcinoma at the transection margin generally warrants additional resection, whereas low-grade dysplastic changes may not justify further sacrifice of pancreatic tissue.

Although current guidelines provide a practical framework for surgical decision-making, their performance remains imperfect. Watanabe et al. demonstrated that the revised 2017 criteria achieve acceptable sensitivity for detecting malignant lesions but remain limited by suboptimal specificity, resulting in a substantial proportion of resections for lesions ultimately found to harbor only low-grade dysplasia [27]. This limitation highlights one of the central dilemmas in IPMN management: balancing the prevention of invasive cancer against the morbidity associated with pancreatic surgery.

The potential benefit of timely intervention was illustrated by Djoumi et al., who reported comparable long-term survival among patients undergoing pre-emptive resection of non-invasive IPMN and those treated for early-stage invasive disease [42]. These findings support the concept that the ideal therapeutic window lies before invasive transformation occurs. At the same time, growing evidence suggests that the traditional binary distinction between surgical and non-surgical management may be overly simplistic. Fong et al. advocated for a more nuanced approach that incorporates biological risk, patient comorbidities, life expectancy, and individual preferences, thereby moving toward a more personalized therapeutic strategy [8].

### 10.2. Postoperative Surveillance

The rationale for postoperative surveillance stems directly from the recognition of IPMN as a diffuse pancreatic field defect rather than a purely localized neoplasm. Consequently, resection of the index lesion does not eliminate the risk of subsequent neoplastic progression within the remnant pancreas.

As discussed previously, Hirono et al. reported an overall recurrence rate of 14.4% in a multicenter cohort of 1074 surgically treated patients, with more than one-third of metachronous high-risk lesions occurring beyond five years after surgery [41]. Importantly, patients who underwent repeat surgery for newly detected high-risk lesions experienced significantly improved survival, emphasizing the clinical value of continued surveillance.

Current surveillance protocols generally recommend cross-sectional imaging every six months during the first postoperative year, followed by annual MRI/MRCP examinations thereafter. In selected patients with resected non-invasive IPMN and prolonged radiological stability, surveillance intervals may be extended. Nevertheless, both the IAP and Kyoto recommendations support lifelong follow-up, reflecting the absence of a clearly defined time point at which the risk of new pancreatic neoplasia can be considered negligible.

Patients with resected invasive IPMN require more intensive monitoring. In addition to imaging of the remnant pancreas, surveillance should include serum CA 19-9 assessment and careful evaluation for distant recurrence. Particular attention should be paid to the lungs, which have been identified as a relatively frequent site of metastatic relapse in several surgical series, including the study by Capretti et al. [43].

### 10.3. Emerging Diagnostic and Therapeutic Technologies

#### Molecular Biomarkers and Liquid Biopsy

One of the most rapidly evolving areas of IPMN research is the development of molecular tools capable of improving risk stratification beyond conventional imaging criteria. The ultimate goal is to identify biomarkers that can reliably distinguish indolent lesions from those destined to progress toward invasive carcinoma.

Several complementary approaches are currently under investigation. Analysis of pancreatic juice using next-generation sequencing has shown particular promise, as the detection of TP53 mutations may provide a non-surgical method for identifying advanced neoplastic progression [17]. Moreover, distinct mutational signatures identified in patients with IPMN-associated PDAC suggest that pancreatic juice profiling may eventually facilitate earlier detection of concomitant pancreatic cancer [19].

Liquid biopsy represents another attractive strategy. Nitschke et al. demonstrated that canonical IPMN-associated mutations, including KRAS and GNAS, can be detected in circulating cell-free DNA and circulating epithelial cells. Notably, differences in GNAS mutation profiles between resected and surveilled patients suggest a potential role for blood-based biomarkers in future risk stratification algorithms [14].

Molecular characterization of cyst fluid continues to advance in parallel. Integrated molecular pathology (IMP) models combine clinical information with genomic analysis of cyst fluid DNA, aiming to improve prediction of malignant transformation. Simpson et al. reported that a simplified IMP model incorporating only main pancreatic duct diameter as a clinical variable achieved improved discrimination of invasive disease [32]. Additional biomarkers, including HMGA2 protein expression, have shown preliminary promise for differentiating low-grade from high-grade dysplasia [33].

Alongside molecular diagnostics, quantitative imaging approaches are gaining increasing attention. Multiparametric prediction models, such as those developed by Park et al., integrate multiple radiological features to estimate malignancy risk and may represent an important step toward data-driven, individualized decision-making [35].

#### Pancreatoscopy

Peroral pancreatoscopy (POP) has emerged as a valuable adjunctive technique for the evaluation of selected IPMNs, particularly those involving the main pancreatic duct. Direct visualization of the ductal epithelium enables more accurate characterization of intraductal lesions, facilitates targeted tissue sampling, and may improve preoperative assessment of disease extent.

Recent technological advances, including improved single-operator platforms and the potential integration of confocal laser endomicroscopy, have further expanded the diagnostic capabilities of pancreatoscopy. Although its precise role remains to be defined, POP may become increasingly important in situations where conventional imaging and EUS provide inconclusive results [34].

### 10.4. Chemoprevention

The concept of chemoprevention in IPMN remains largely theoretical, but growing interest has focused on the possibility of modifying disease progression through pharmacological intervention. To date, however, no agent has demonstrated sufficient evidence to support routine clinical use.

Among the available data, Takasaki et al. reported that low-dose aspirin was not associated with a significant reduction in pancreatic cancer incidence among patients with IPMN. Nevertheless, aspirin users exhibited a lower frequency of progressive main pancreatic duct dilation, raising the possibility that anti-inflammatory therapies may influence disease evolution even in the absence of a measurable effect on cancer incidence [48].

Other agents, including metformin, statins, and non-steroidal anti-inflammatory drugs, have also been proposed as potential chemopreventive strategies based on epidemiological and mechanistic observations. However, none have yet been validated in prospective studies specifically designed for IPMN populations. Consequently, chemoprevention should currently be regarded as an important area of future investigation rather than an established component of clinical management.

### 10.5. Future Perspectives

**Figure 5:**
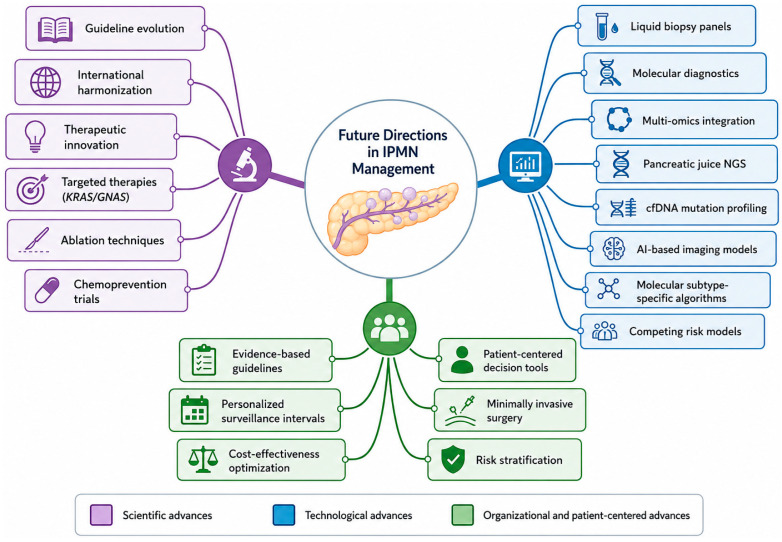
Future directions in the management of intraductal papillary mucinous neoplasms (IPMNs). Future progress in IPMN management will rely on the integration of three interconnected pillars: scientific advances, technological innovation, and organizational/patient-centered approaches. Improvements in molecular understanding, therapeutic development, biomarker discovery, artificial intelligence, multi-omics profiling, personalized surveillance, and evidence-based clinical decision-making are expected to refine risk stratification, optimize treatment selection, and enable increasingly individualized management of patients with IPMN. NGS, next-generation sequencing; cfDNA, circulating cell-free DNA.

The future of IPMN management is likely to be defined by the convergence of advances in molecular diagnostics, quantitative imaging, therapeutic innovation, and patient-centered decision-making (Figure 5). Collectively, these developments are driving a transition from the current morphology-based paradigm toward a more personalized and biologically informed approach to risk assessment and treatment.

One of the most important anticipated advances is the integration of molecular information into routine clinical decision-making. Although current management algorithms rely predominantly on radiological features such as cyst size, mural nodules, and main pancreatic duct dilation, growing evidence indicates that genomic, transcriptomic, and proteomic profiling may provide a more accurate representation of biological risk. Molecular alterations involving KRAS, GNAS, TP53, and SMAD4, together with subtype-specific developmental pathways, offer the potential to identify lesions that are truly destined for malignant progression while avoiding overtreatment of biologically indolent neoplasms [10,17,21].

Parallel advances in diagnostic technology are expected to further refine risk stratification. Liquid biopsy approaches, including circulating cell-free DNA analysis and molecular characterization of pancreatic juice, may enable non-invasive detection of high-risk lesions and concomitant pancreatic cancer. At the same time, artificial intelligence and machine learning algorithms applied to cross-sectional imaging, endoscopic ultrasound, and molecular datasets are increasingly capable of integrating complex multimodal information into predictive models that exceed the performance of individual clinical variables. Such approaches may ultimately replace the current reliance on isolated morphological thresholds with dynamic, individualized risk prediction systems.

Therapeutic innovation represents another promising frontier. Increasing understanding of the molecular pathways underlying IPMN progression has generated interest in targeted interventions directed against key oncogenic drivers, including KRAS-associated signaling networks and the GNAS–cAMP–PKA pathway. Although these strategies remain investigational, the success of molecularly targeted therapies in other malignancies provides proof of principle that pharmacological interruption of the dysplasia–carcinoma sequence may eventually become feasible. Similarly, minimally invasive approaches such as EUS-guided radiofrequency ablation and cyst ablation techniques are being explored as potential alternatives to surgery for carefully selected patients who are poor operative candidates.

Beyond technological innovation, future progress will require the refinement and harmonization of clinical practice guidelines. Persistent discrepancies among international recommendations continue to generate variability in surveillance and treatment decisions, reflecting the limited availability of high-quality prospective data. The Kyoto 2024 guidelines represent an important step toward updating risk stratification strategies, but further progress will depend on large prospective registries, multicenter collaborations, and ideally randomized studies capable of providing stronger evidence for clinical decision-making [7,8,38].

Perhaps most importantly, the next generation of IPMN management will need to place greater emphasis on patient-centered care. As highlighted throughout this review, many patients with IPMN are elderly and frequently face competing health risks that may exceed their likelihood of pancreatic cancer-related mortality. Consequently, surveillance and treatment decisions should increasingly incorporate life expectancy, comorbidity burden, quality-of-life considerations, and patient preferences alongside conventional oncological endpoints. The development of shared decision-making tools capable of communicating individualized risk in a clear and accessible manner will therefore represent a critical component of future clinical practice.

Ultimately, the goal of future IPMN management is not simply earlier detection of malignancy, but the creation of an integrated precision-medicine framework that combines molecular biology, advanced imaging, clinical risk assessment, and patient-specific factors to deliver the right intervention to the right patient at the right time.

## 11. Conclusions

Intraductal papillary mucinous neoplasm (IPMN) has undergone a remarkable conceptual evolution since its initial description in the 1980s as a poorly defined mucin-producing pancreatic lesion. Over the past decades, advances in pathology, molecular biology, imaging, and clinical management have transformed IPMN into one of the most extensively studied precursor lesions in gastroenterology and pancreatic oncology.

Among the most important advances has been the recognition that IPMN follows a defined neoplastic progression pathway characterized by the sequential accumulation of molecular alterations. Likewise, the four histological subtypes are now understood to represent biologically distinct entities with unique molecular signatures, patterns of progression, and prognostic implications rather than simple morphological variants. The concept of IPMN as a pancreatic field defect has also emerged as a central paradigm, supported by molecular evidence demonstrating that metachronous lesions and concomitant pancreatic ductal adenocarcinomas may arise from clonally distinct lineages within the same gland.

At the same time, several widely held assumptions continue to be challenged. Despite significant advances in imaging and risk stratification, current guideline-based algorithms remain imperfect, and a substantial proportion of lesions resected because of suspected high-risk features ultimately prove to harbor only low-grade dysplasia. Similarly, the optimal duration and intensity of surveillance remain subjects of ongoing debate. Although lifelong follow-up has traditionally been recommended, emerging evidence and the 2024 Kyoto guidelines suggest that surveillance discontinuation may be appropriate in carefully selected patients with small, stable BD-IPMNs, underscoring the need for individualized management.

Perhaps the most important message emerging from contemporary IPMN research is that management can no longer rely exclusively on a morphology-based approach. The traditional paradigm, largely centered on cyst size and ductal dilation thresholds, is progressively giving way to a more nuanced model that integrates radiological findings with molecular characteristics, patient comorbidities, life expectancy, competing mortality risks, and individual preferences. This transition mirrors the broader evolution of modern oncology toward precision medicine and patient-centered care.

Looking ahead, the convergence of molecular diagnostics, liquid biopsy technologies, artificial intelligence, advanced imaging analytics, and large prospective registries has the potential to transform IPMN management from a predominantly consensus-based discipline into a truly evidence-based and personalized field.

Viewed through the lens of its history, the evolution of IPMN research illustrates how scientific progress transforms clinical myths into biological realities, and several assumptions that once dominated clinical practice have now been definitively revised.

In this sense, the future of IPMN management lies not in choosing between surveillance and surgery but in developing integrated precision-medicine frameworks capable of delivering the right intervention to the right patient at the right time. As molecular diagnostics, artificial intelligence, and personalized risk assessment become increasingly integrated into clinical practice, many of today’s myths may become tomorrow’s realities.

## Figures and Tables

**Table 1:** Pancreatic diseases that should be considered in differential diagnosis with IPMN.

Entity	Key Distinguishing Features
Mucinous cystic neoplasm (MCN)	Ovarian-type stroma (pathognomonic); no ductal communication; almost exclusively in women (>95%); body/tail location; typically unilocular or oligolocular; does not communicate with the MPD on MRCP
Serous cystadenoma (SCN)	Non-mucinous; microcystic (“honeycomb”) pattern with central stellate scar; VHL gene-driven; benign; low CEA in cyst fluid; predominantly in women
Pancreatic pseudocyst	History of pancreatitis or trauma; no epithelial lining; inflammatory fibrous wall; elevated amylase; low CEA
Intraductal tubulopapillary neoplasm (ITPN)	Tubular (rather than papillary) architecture; rare; may lack GNAS mutations; tends to have dense cellular proliferation without mucin overproduction
Solid pseudopapillary neoplasm (SPN)	Young women; solid and cystic with hemorrhagic degeneration; β-catenin mutations; low malignant potential
PanIN	Microscopic (<5 mm); flat or papillary; shares KRAS but typically lacks GNAS; cannot be detected by imaging
PDAC with cystic degeneration	Solid mass with secondary cystic change; aggressive features; heterogeneous enhancement
Cystic neuroendocrine tumor	Enhancing rim; chromogranin/synaptophysin positive; characteristic imaging features

**Table 2:** Surgical approach according to different clinical presentation of IPMN.

Clinical Scenario	Recommended Procedure
IPMN of the pancreatic head with high-risk features	Pancreatoduodenectomy (Whipple procedure)
IPMN of the body/tail with high-risk features	Distal pancreatectomy ± splenectomy
Diffuse main-duct involvement	Total pancreatectomy (selected cases)
Multifocal BD-IPMN without high-risk features	Surveillance preferred over surgery
Borderline surgical candidates	Individualized decision balancing oncological risk vs. operative risk

## Data Availability

No new data were created or analyzed in this study.

## References

[1] Fong Z.V., Fernández-del Castillo C. (2016). Intraductal papillary mucinous neoplasms of the pancreas. Surg. Clin. N. Am..

[2] Tanaka M., Fernández-del Castillo C., Kamisawa T., Jang J.Y., Levy P., Ohtsuka T., Salvia R., Shimizu Y., Tada M., Wolfgang C.L. (2017). Revisions of international consensus Fukuoka guidelines for the management of IPMN of the pancreas. Pancreatology.

[3] McCarty T.R., Njei B. (2016). Trends in malignant intraductal papillary mucinous neoplasms in United States adults, 1990–2010: A SEER database analysis. Gastroenterol. Rep..

[4] Chelliah A., Kalimuthu S., Chetty R. (2016). An overview of intraductal tubular neoplasms of the pancreas. Ann. Diagn. Pathol..

[5] Weissman S., Thaker R.K., Zeffren N., Sarfaraz R., Dedousis J. (2019). Intraductal papillary mucinous neoplasm of the pancreas: Understanding the basics and beyond. Cureus.

[6] Park J.K., Hwang J.W. (2023). Research trends and future directions of intraducttal papillary mucinous neoplasms: A bibliometric and visualization analysis of more than 30 years of research. Medicine.

[7] Levink I.J.M., Bruno M.J., Cahen D.L. (2018). Management of intraductal papillary mucinous neoplasms: Controversies in guidelines and future perspectives. Curr. Treat. Options Gastroenterol..

[8] Fong Z.V., Hernandez-Barco Y.G., Fernández-del Castillo C. (2022). Clinical guidelines for the management of intraductal papillary mucinous neoplasms: The need for a more graded approach to clinical decisions. J. Gastrointest. Surg..

[9] Tanaka M., Fernández-del Castillo C., Adsay V., Chari S., Falconi M., Jang J.-Y., Kimura W., Levy P., Pitman M.B., Schmidt C.M. (2012). International consensus guidelines 2012 for the management of IPMN and MCN of the pancreas. Pancreatology.

[10] (2018). The European Study Group on Cystic Tumours of the Pancreas. European evidence-based guidelines on pancreatic cystic neoplasms. Gut.

[11] Mayhew M.M., Buerlein R.C.D., Zaydfudim V.M. (2025). Current considerations for non-operative surveillance of intraductal papillary mucinous neoplasms. Semin. Liver Dis..

[12] Furukawa T., Kuboki Y., Tanji E., Yoshida S., Hatori T., Yamamoto M., Shibata N., Shimizu K., Kamatani N., Shiratori K. (2011). Whole-exome sequencing uncovers frequent GNAS mutations in intraductal papillary mucinous neoplasms of the pancreas. Sci. Rep..

[13] Wu J., Matthaei H., Maitra A., Dal Molin M., Wood L.D., Eshleman J.R., Goggins M., Canto M.I., Schulick R.D., Edil B.H. (2011). Recurrent GNAS mutations define an unexpected pathway for pancreatic cyst development. Sci. Transl. Med..

[14] Hu Y., Jones D., Esnakula A.K., Krishna S.G., Chen W. (2024). Molecular pathology of pancreatic cystic lesions with a focus on malignant progression. Cancers.

[15] Nitschke C., Tölle M., Walter P., Meißner K., Goetz M., Kropidlowski J., Berger A.W., Izbicki J.R., Nickel F., Hackert T. (2024). KRAS and GNAS mutations in cell-free DNA and circulating epithelial cells in patients with intraductal papillary mucinous neoplasms—An observational pilot study. Mol. Oncol..

[16] Gaujoux S., Pasmant E., Silve C., Mehsen-Cetre N., Coriat R., Rouquette A., Douset B., Prat F., Leroy K. (2019). McCune-Albright syndrome as a genetic predisposition to IPMN-associated pancreatic cancer. Medicine.

[17] Takano S., Fukasawa M., Kadokura M., Shindo H., Takahashi E., Hirose S., Maekawa S., Mochizuki K., Kawaida H., Itakura J. (2017). Next-generation sequencing revealed TP53 mutations to be malignant marker for intraductal papillary mucinous neoplasms that could be detected using pancreatic juice. Pancreas.

[18] Chang X.Y., Wu Y., Li Y., Wang J., Chen J. (2018). Intraductal papillary mucinous neoplasms of the pancreas: Clinical association with KRAS. Mol. Med. Rep..

[19] Takano S., Fukasawa M., Kadokura M., Shindo H., Takahashi E., Hirose S., Fukasawa Y., Kawakami S., Hayakawa H., Maekawa S. (2019). Mutational patterns in pancreatic juice of patients with intraductal papillary mucinous neoplasm and concomitant pancreatic ductal carcinoma. Pancreas.

[20] Collet L., Ghurburrun E., Meyers N., Assi M., Pirlot B., Leclercq I.A., Couvelard A., Komuta M., Cros J., Demetter P. (2020). Kras and Lkb1 mutations synergistically induce intraductal papillary mucinous neoplasm derived from pancreatic duct cells. Gut.

[21] Kobayashi T., Omori Y., Ono Y., Karasaki H., Mizukami Y., Makino N., Motoi F., Unno M., Ueno Y., Furukawa T. (2021). Developmental pathways of multiple types of intraductal papillary mucinous neoplasm of the pancreas. J. Gastroenterol..

[22] Omori Y., Ono Y., Tanino M., Karasaki H., Yamaguchi H., Furukawa T., Enomoto K., Ueda J., Sumi A., Katayama J. (2019). Pathways of Progression From Intraductal Papillary Mucinous Neoplasm to Pancreatic Ductal Adenocarcinoma Based on Molecular Features. Gastroenterology.

[23] Pflüger M.J., Griffin J.F., Hackeng W.M., Kawamoto S., Yu J., Chianchiano P., Shin E., Lionheart G., Tsai H.-L., Wang H. (2022). The impact of clinical and pathological features on IPMN recurrence after surgical resection. Ann. Surg..

[24] Matsuzaka S., Karasaki H., Ono Y., Ogata M., Oikawa K., Tamakawa S., Chiba S.-I., Muraki M., Yokochi T., Funakoshi H. (2016). Tracing clonal evolution of adenosquamous carcinoma arising from intraductal papillary mucinous neoplasm. Pancreas.

[25] Aronsson L., Andersson R., Ansari D. (2017). Intraductal papillary mucinous neoplasm of the pancreas—Epidemiology, risk factors, diagnosis, and management. Scand. J. Gastroenterol..

[26] Huang X., Zhang B., Zhao J., Sun C., Kong K., Deng L., Liu Y., Zheng J. (2019). Increased risk of second primary cancers following diagnosis of malignant intraductal papillary mucinous neoplasm: A population-based study. Front. Oncol..

[27] Watanabe Y., Endo S., Nishihara K., Ueda K., Mine M., Tamiya S., Nakano T., Tanaka M. (2018). The validity of the surgical indication advocated by the 2017 revised International Association of Pancreatology consensus guidelines for IPMN. Surg. Today.

[28] de Pretis N., Martinelli L., Amodio A., Caldart F., Crucillà S., Battan M.S., Zorzi A., Crinò S.F., Conti Bellocchi M.C., Bernardoni L. (2025). Branch Duct IPMN-Associated Acute Pancreatitis in a Large Single-Center Cohort Study. Diagnostics.

[29] Ma G., Li G., Xiao Z., Gou A., Xu Y., Song S., Guo K., Liu Z. (2021). A narrative review of IPMN: Pathogenesis, diagnosis, and treatment of a true precancerous lesion. Gland Surg..

[30] Min J.H., Kim Y.K., Kim S.K., Kim H., Ahn S. (2021). Intraductal papillary mucinous neoplasm of the pancreas: Diagnostic performance of the 2017 international consensus guidelines using CT and MRI. Eur. Radiol..

[31] Tacelli M., Celsa C., Magro B., Barchiesi M., Barresi L., Capurso G., Arcidiacono P.G., Cammà C., Crinò S.F. (2020). Diagnostic performance of endoscopic ultrasound through-the-needle microforceps biopsy of pancreatic cystic lesions: Systematic review with meta-analysis. Dig. Endosc..

[32] Simpson R.E., Cockerill N.J., Yip-Schneider M.T., Ceppa E.P., House M.G., Zyromski N.J., Nakeeb A., Al-Haddad M.A., Schmidt C. (2019). Clinical criteria for integrated molecular pathology in intraductal papillary mucinous neoplasm: Less is more. HPB.

[33] DiMaio C.J., Weis-Garcia F., Bagiella E., Tang L.H., Allen P.J. (2016). Pancreatic cyst fluid concentration of HMGA2 protein acts as a differential biomarker of dysplasia in IPMN. Gastrointest. Endosc..

[34] de Jong D.M., Stassen P.M.C., Groot Koerkamp B., Molenaar I.Q., Busch O.R., Besselink M.G., Fockens P., van Hooft J.E., van Dieren S., Del Chiaro M. (2022). The role of pancreatoscopy in the diagnostic work-up of IPMN: A systematic review and meta-analysis. Endoscopy.

[35] Park J., Kim J.H., Bae J.S., Lee J., Kim S.H., Kim S.Y., Park S.H., Kim H., Choi J.-Y., Lee S.S. (2024). An imaging-based model for predicting the malignancy risk of intraductal papillary mucinous neoplasm. Eur. Radiol..

[36] Hasegawa H., Fukasawa M., Takano S., Kawakami S., Kuratomi N., Harai S., Yoshimura D., Imagawa N., Okuwaki T., Kuno T. (2024). New surgical criteria for IPMN based on the age-adjusted Charlson comorbidity index and presence of solid component. Diagnostics.

[37] Del Chiaro M., Verbeke C. (2017). Intraductal papillary mucinous neoplasms of the pancreas: Reporting clinically relevant features. Histopathology.

[38] Marchegiani G., Pollini T., Burelli A., Han Y., Jung H.S., Kwon W., Rocha Castellanos D.M., Crippa S., Belfiori G., Arcidiacono P.G. (2023). Surveillance for Presumed BD-IPMN of the Pancreas: Stability, Size, and Age Identify Targets for Discontinuation. Gastroenterology.

[39] Borges M.P.F., Rebelo P., Vilas-Boas F., Graça L., Carneiro S., Bouça-Machado T. (2025). Assessing the Accuracy of the International Evidence-Based Kyoto Guidelines for Detecting Malignancy in Intraductal Papillary Mucinous Neoplasms of the Pancreas. GE Port. J. Gastroenterol..

[40] Sekine K., Nagata N., Hisada Y., Yamamoto K., Mukai S., Tsuchiya T., Machitori A., Kojima Y., Yada T., Yamamoto N. (2024). Identifying predictors for comorbidities-related mortality versus pancreatic cancer-related mortality in patients with IPMN: A multicenter long-term follow-up. United Eur. Gastroenterol. J..

[41] Hirono S., Shimizu Y., Ohtsuka T., Kin T., Hara K., Kanno A., Koshita S., Hanada K., Kitano M., Inoue H. (2020). Recurrence patterns after surgical resection of IPMN of the pancreas: A multicenter retrospective study of 1074 patients by the Japan Pancreas Society. J. Gastroenterol..

[42] Djoumi Y., Sadr-Azodi O., Vujasinovic M., Del Chiaro M., Sparrelid E., Ghorbani P., Holmberg M. (2023). Pre-emptive resection for intraductal papillary mucinous neoplasia—Long-term outcome is similar between non-invasive and early invasive lesions. Pancreatology.

[43] Capretti G., Nebbia M., Gavazzi F., Nappo G., Ridolfi C., Sollai M., Spaggiari P., Bozzarelli S., Carrara S., Luberto A. (2022). Invasive IPMN relapse later and more often in lungs in comparison to pancreatic ductal adenocarcinoma. Pancreatology.

[44] Suraju M.O., Snow A., Nayyar A., Chang J., Sherman S.K., Hoshi H., Howe J.R., Chan C.H. (2023). Peritoneal metastases after intraductal papillary mucinous neoplasm resection: How common are they?. J. Surg. Res..

[45] Marsoner K., Haybaeck J., Csengeri D., Waha J.E., Schagerl J., Langeder R., Mischinger H.J., Kornprat P. (2016). Pancreatic resection for intraductal papillary mucinous neoplasm—A thirteen-year single-center experience. BMC Cancer.

[46] Aronsson L., Ansari D., Andersson B., Persson U., Fridhammar A., Andersson R. (2018). Intraductal papillary mucinous neoplasms of the pancreas—A cost-effectiveness analysis of management strategies for the branch-duct subtype. HPB.

[47] Caringi S., Delvecchio A., Casella A., Ferraro V., Stasi M., Tralli N., Manzia T.M., Tedeschi M., Memeo R. (2026). Parenchyma-Sparing Pancreatic Surgery: Current Indications, Results, and Future Prospects. Cancers.

[48] Takasaki Y., Nagata N., Imbe K., Hisada Y., Sekine K., Tajima T., Yanase M., Fujimoto K., Akiyama J., Uemura N. (2017). Effect of low-dose aspirin use on pancreatic cancer development and morphological changes on imaging in IPMN: A long-term cohort study. United Eur. Gastroenterol. J..

